# Evaluating the potential of (epi)genotype-by-low pass nanopore sequencing in dairy cattle: a study on direct genomic value and methylation analysis

**DOI:** 10.1186/s40104-023-00896-3

**Published:** 2023-07-12

**Authors:** Oscar González-Recio, Adrián López-Catalina, Ramón Peiró-Pastor, Alicia Nieto-Valle, Monica Castro, Almudena Fernández

**Affiliations:** 1 Dpt. Mejora Genética Animal, INIA-CSIC, Ctra La Coruña Km 7.5, 28040 Madrid, Spain; 2 ETSIAAB, Universidad Politécnica de Madrid. Ciudad Universitaria S/N, 28040 Madrid, Spain

**Keywords:** Genomic selection, Genomic values, Low pass sequencing, Low sequencing imputation, Polygenic risk score

## Abstract

**Background:**

Genotype-by-sequencing has been proposed as an alternative to SNP genotyping arrays in genomic selection to obtain a high density of markers along the genome. It requires a low sequencing depth to be cost effective, which may increase the error at the genotype assigment. Third generation nanopore sequencing technology offers low cost sequencing and the possibility to detect genome methylation, which provides added value to genotype-by-sequencing. The aim of this study was to evaluate the performance of genotype-by-low pass nanopore sequencing for estimating the direct genomic value in dairy cattle, and the possibility to obtain methylation marks simultaneously.

**Results:**

Latest nanopore chemistry (LSK14 and Q20) achieved a modal base calling accuracy of 99.55%, whereas previous kit (LSK109) achieved slightly lower accuracy (99.1%). The direct genomic value accuracy from genotype-by-low pass sequencing ranged between 0.79 and 0.99, depending on the trait (milk, fat or protein yield), with a sequencing depth as low as 2 × and using the latest chemistry (LSK114). Lower sequencing depth led to biased estimates, yet with high rank correlations. The LSK109 and Q20 achieved lower accuracies (0.57–0.93). More than one million high reliable methylated sites were obtained, even at low sequencing depth, located mainly in distal intergenic (87%) and promoter (5%) regions.

**Conclusions:**

This study showed that the latest nanopore technology in useful in a LowPass sequencing framework to estimate direct genomic values with high reliability. It may provide advantages in populations with no available SNP chip, or when a large density of markers with a wide range of allele frequencies is needed. In addition, low pass sequencing provided nucleotide methylation status of > 1 million nucleotides at ≥ 10 × , which is an added value for epigenetic studies.

## Introduction

Advances in genotyping platforms over the past two decades have enabled the prediction of genetic value in individuals for the implementation of genomic selection in animal and plant populations [1]. They also allowed the prediction of polygenic risk scores in human populations that predict the probability of suffering certain diseases [2]. Initially, genotyping arrays consisted of a few hundreds or thousands of SNPs, but improvements in the technology soon after allowed for the incorporaion of hundreds of thousands of SNPs in genotyping arrays. Methods for genotype imputation have also contributed to the use of different genotyping platforms or different densities of genotyping arrays [3, 4]. A major disadvantage of SNP arrays is that their design is often based on few animals/populations, which limits their use in other populations not considered in the design. In addition, low frequency and rare variants are seldom included in the genotyping arrays, which may miss linkage disequilibrium with relevant causal variants of certains diseases and traits. More recently, genotype-by-sequencing has allowed capturing millions of variants along the genome [5]. Genotype-by-sequencing techniques can be used to align DNA reads against a reference genome and detect polymorphic positions with bioinformatics tools throughout the genome, regardless of whether they have been previously detected or included in an array design. The precision of this genotype-by-sequencing is mainly determined by the sequencing depth. However, the limitations in precision at low sequencing depth can be compensated for by imputation strategies, as its more affordable cost allows sequencing many more individuals in the population, improving the statistical power of genomic selection and genomic studies [6]. Detecting a larger number of variants at different minor allele frequencies helps to discover association signals in genome-wide association studies and estimate the genetic value of individuals with a similar precision as SNP chips [7, 8]. Genetic imputation has also been applied to genotype-by-sequencing data, which needs to deal with artifact errors due to low depth or low-pass sequencing (LPS). Some methods have already been proposed to palliate this limitation [6, 9–11].

Third-generation sequencing techniques such as Oxford Nanopore Techonology (ONT) have been explored as an option for genomic selection using information at low sequencing depth [12]. This technique allows for fast and low-cost sequencing at the expense of a higher error rate compared to Sanger or sequencing by synthesis. However, the latest nanopore chemistry offers higher accuracy which may increase the accuracy of the prediction of genetic values using this technique. Additionally, nanopore sequencing can simultaneously detect epigenetic modifications at the nucleotide level, and it is obtained at no additional cost. This information can be used in breeding programs and epigenetic studies in livestock and plants [13].

Nanopore sequencing has already been used for pathogen identification, metagenomic studies, and the assembly of reference genomes. However, its higher sequencing error rate has discouraged its use for predicting the genetic value of individuals. Since the accuracy and yield of the technique has improved in recent years, along with its low cost, better portability, the ability to obtain modified bases, and specific bioinformatics tools, it is now more attractive for exploring its performance in genomic prediction under a genotype-by-low pass sequencing framework that includes epigenetic information. It is also an alternative tool to genomic research involving epigenetics.

The aim of this study was to determine the accuracy of epi-genotype low pass sequencing (EpiGLow) using Nanopore Technology in terms of basecalling, imputation, and prediction of genetic merit, in comparison to SNP genotyping arrays within a genomic selection framework. Both older and more recent nanopore chemistries were compared, and the potential to include epigenetic information was also evaluated.

## Materials and methods

### Samples and DNA extraction

Blood samples were obtained from 32 Holstein female calves during routine practices in a commercial farm of 1,000 lactating cows in the Northeast regions of Spain. The calves were born in the same year-season and were daughters of 8 different sires. These samples were obtained by a veterinarian during the routinary process for genomic evaluations within the official Holstein breeding program in Spain (https://www.conafe.com). One sample from each animal was sent to the official genotyping lab, and was genotyped using the Illumina EUROG MD genotyping microarray that contains approximately 62,000 markers. Another sample was sent to the department of animal breeding at INIA-CSIC, where DNA was extracted using the Monarch® HMW DNA Extraction Kit for Cells Blood (New England BioLabs, Ipswich, MA, USA). This DNA was then prepared for sequencing.

### Sequencing

The purified DNA was sequenced in either a MinION Mk1B or GridION X5 Mk1 from Oxford Nanopore Technologies (ONT) (Oxford, UK). The individual DNA libraries were prepared starting with 3 µg of DNA, and then following the manufacturer recommendations. Twelve samples were sequenced using the kit SQK-LSK109 (LSK109) in R9.4 flow cells, multiplexing 6 samples per flow cell. Other twelve samples were sequenced using the kit SQK-LSK110 (Q20) in R9.4 flow cells, also multiplexing 6 samples per flow cell. This kit uses a motor protein with a slower translocation speed through the nanopore, which increases the basecalling accuracy. Finally, the remaining six samples were sequenced following the protocol from the kit SQK-LSK114 (LSK114) in R10.4.1 flow cells, multiplexing 2 samples per flow cell. This kit used an improved motor protein and a wider nanopore type. Two samples from LSK109 were discarded for not yielding enough reads to start the bioinformatic analyses. The samples were intented to be as balanced as possible according to sire and kit, with representation of the sires with more than one daughter in all kits.

### Bioinformatic pipeline

Basecalling was performed with guppy toolkit version 6.4.2 using SUP mode. Reads with length ≤ 150 bp or ONT quality score < 10 were discarded. Remaining reads were aligned against *Bos taurus* reference genome (ARS-UCD1.2) using minimap2 aligner, with option -ax map-ont [14], a general-purpose alignment program to map DNA or long mRNA sequences. Coverage statistics were calculated with samtools coverage [15]. After the alignment, the content and percentage of mismatches by read were computed. The CIGAR string samtools and the edit distance from the reference or number of mismatches per pair (NM_tag value_) from the alignment were used to extract the total length of insertions and deletions and single nucleotides mismatches for each read. The NM_tag value_ is the sum of total mismatch score (TMS) and length of insertions and deletions. Thus, TMS was computed as:

1 $$TMS=NM_{tagvalue}-\mathit(length\;of\;insertions\mathit)\mathit\;\mathit-\mathit\;\mathit(length\;of\;deletions\mathit)$$

The accuracy of each read was then calculated as:

2 $$Accuracy=1-\frac{TMS}{readlength}$$

Then, variants were called using Clair3 v0.1-r11 [16]. Variants with sequencing depth ≤ 2 were discarded for downstream analysis unless the variant was equal to the alternate allele in the 1,000 bull genomes reference population. A heterozygous position was called if the allele frequency was larger than 0 and lower than 90%. The resulting variants were then imputed to whole genome sequencing using the 1000 Bull Genomes (Run 6) Project [17] and Beagle version 5.2 [18], using the Holstein reference population (844 animals) as reference. We kept those common variants (38,747) in the Illumina Bovine50K beadchip that were included in the official genomic evaluations of milk yield (MY), fat yield (FY) and protein yield (PY) from the Spanish Holstein Association (CONAFE). Accuracy of imputation was evaluated as the mismatch rate between LPS and SNP genotypes.

### Computing direct genomic values

Direct genomic values (DGV_it_) for each individual *i* and trait (*t* = MY, FY, PY) (either from SNP beadchips or LPS) were calculated as:

3 $${DGV}_{it}={\mu }_{t}+\sum_{j=1}^{p}{x}_{j}{\beta }_{jt}$$

where *µ*_*t*_ is some intercept value specific for each trait, *x*_*j*_ is either the SNP genotype or the dosage allele (DA) from imputed ONT sequencing, and β_*jt*_ is the allele substitution effect for SNP*j* and trait *t*, provided by CONAFE. The closeness between DGVs estimated from LPS and SNP chips was evaluated through the *R*^2^ obtained from regressing DVGs from SNP chips (as benchmark) on DGVs obtained from LPS. The intercept and slope of the linear regression were also evaluated.

### Detection of modified based

Modified bases (5mC) were extracted from samples sequenced with LSK114 kit. Methylation marks were detected from bam files produced by the built-in GridION MinKnow basecaller (version 22.12.5) using modbam2bed tool provided by Nanopore software [19]. Genetic features and coordinates were annotated using the R package ChIPseeker [20]. Promoter regions were called using the function getPromoter using the transcription annotated genome for *Bos taurus* and the annotation package org.Bt.eg.db. The transcription start site (TSS) region was defined as −3,000 to 3,000 base pairs from the transcription start site. Sequencing depth thresholds of 4 × , 7 × and 10 × were compared to determine the variation in the genetic features lost when establishing a more stringent filter. The genetic feature in which the methylation marks are located were called using the plotAnnoBar function. Then, heatmaps depicting the distribution of methylation marks in the promoter regions were obtained using the tagHeatmap function.

## Results

### Descriptive summary

**Fig. 1:**
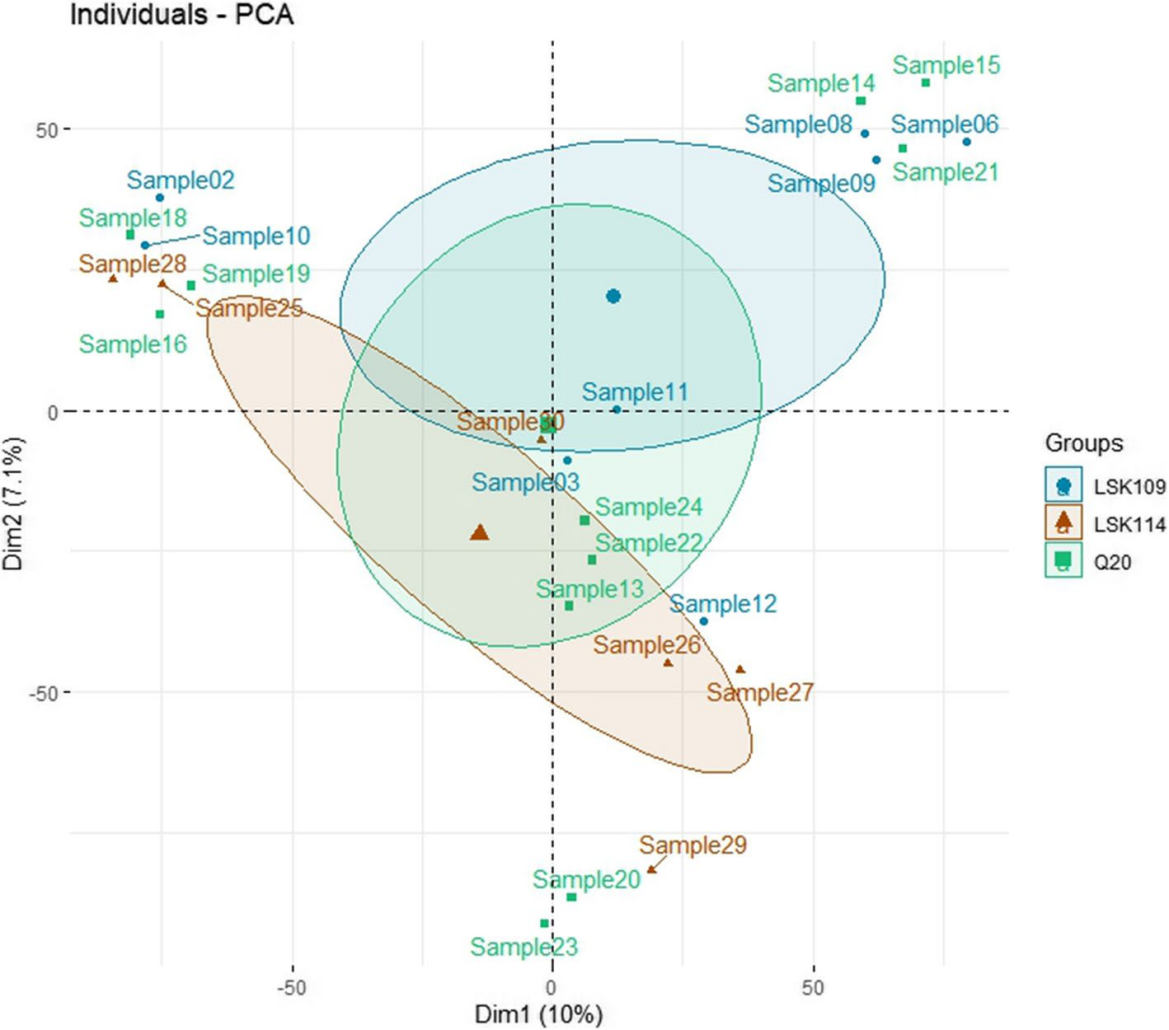
Principal component analysis plot based on the genotypoes from the SNP chips using the first and second principal components. The samples are grouped by kit, showing no clusterization depending on the genomic background of the samples

A summary of the samples kept after quality control is shown in Table 1. The kit LSK109 showed higher yield than Q20, which translated into a higher average sequencing depth (0.6 × vs. 0.4 ×) and a larger number of called variants (221 k vs. 142 k). Samples sequenced with the LSK114 kit showed a higher average sequencing depth (2.1 ×  ± 0.4 × SD) and a larger initial number of variants (1,635 k ± 455 k SD). Improved yield from LSK114 was partially determined because only two samples were multiplexed per flowcell. However, it is equivalent to a 0.8 × sequencing depth if six samples per flow cell would have been multiplexed as in LSK109 and Q20 kits. This circumstance is evaluated below to evaluate LSK114 under lower sequencing depth. The samples did not show any clusterization according to genetic background and kit, based on a PCA plot from the SNP chips genotypes (Fig. 1). The ancestry of the samples are not expected to have a relevant impact on the results obtained from the downstream analyses.

**Table 1:** Summary information for the samples sequenced including kit, sequencing depth, genome coverage and number of variants detected after filtering

Sample	Kit	Sequencing depth	Coverage	Number of variants
Sample 1	LSK109	0.36	27	69,700
Sample 2	LSK109	0.51	36	139,412
Sample 3	LSK109	0.41	29	84,872
Sample 5	LSK109	0.54	37	143,414
Sample 6	LSK109	0.54	37	151,828
Sample 8	LSK109	0.61	41	169,916
Sample 9	LSK109	0.45	32	108,916
Sample 10	LSK109	1.04	58	495,019
Sample 11	LSK109	0.88	53	361,627
Sample 12	LSK109	1.07	60	490,223
Sample 13	Q20	0.41	29	115,022
Sample 14	Q20	0.30	22	65,840
Sample 15	Q20	0.38	27	93,316
Sample 16	Q20	0.31	22	67,232
Sample 17	Q20	0.33	24	76,340
Sample 18	Q20	0.36	26	91,323
Sample 19	Q20	0.86	49	419,787
Sample 20	Q20	0.47	32	145,412
Sample 21	Q20	0.74	44	321,066
Sample 22	Q20	0.31	22	74,607
Sample 23	Q20	0.49	30	140,729
Sample 24	Q20	0.38	26	94,729
Sample 25	LSK114	2.43	82	1,924,878
Sample 26	LSK114	2.10	76	1,533,807
Sample 27	LSK114	1.87	73	1,311,229
Sample 28	LSK114	1.92	73	1,375,101
Sample 29	LSK114	1.78	71	1,246,664
Sample 30	LSK114	2.93	87	2,420,455

### Variant calling accuracy

**Fig. 2:**
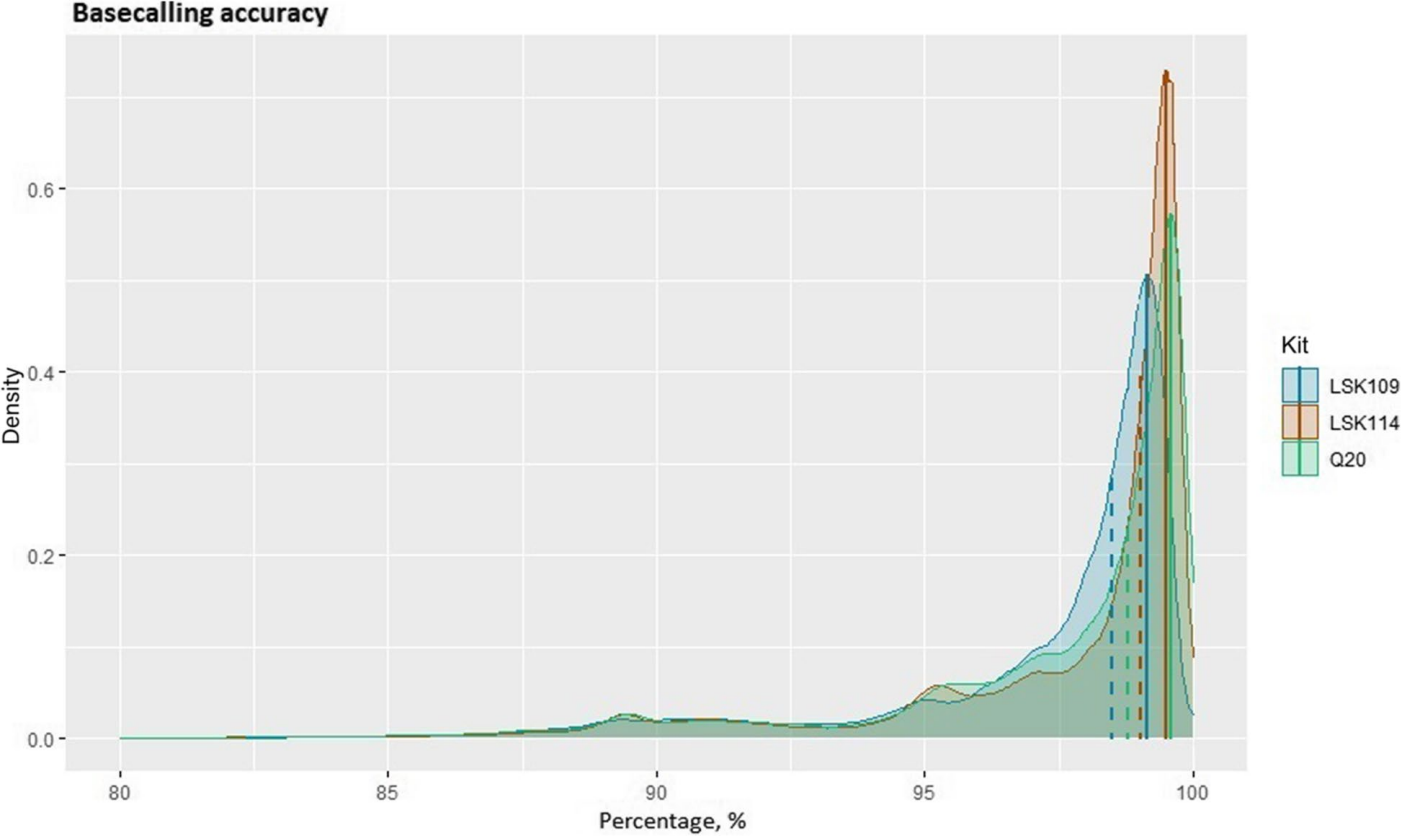
Density plot of the basecalling accuracy for each sequencing analysed, measured as Equation (2). Mode value from each kit is depicted as a vertical solid line. Median value from each kit is depicted as a dashed vertical line

Basecalling accuracy from each sequencing kit is depicted in Fig. 2. Median accuracy was 98.5%, 98.7%, and 99.0% for the LSK109, Q20, and LSK114 kits, respectively. Mode accuracy was 99.1%, 99.6%, and 99.5% for the LSK109, Q20, and LSK114 kits, respectively. It must be noted that this is a down-limit accuracy because it was calculated against the reference genome, so true variants are incorrectly counted as errors. Nonetheless, a significant number of reads showed basecalling accuracies below 95%.

### Imputation accuracy

**Fig. 3:**
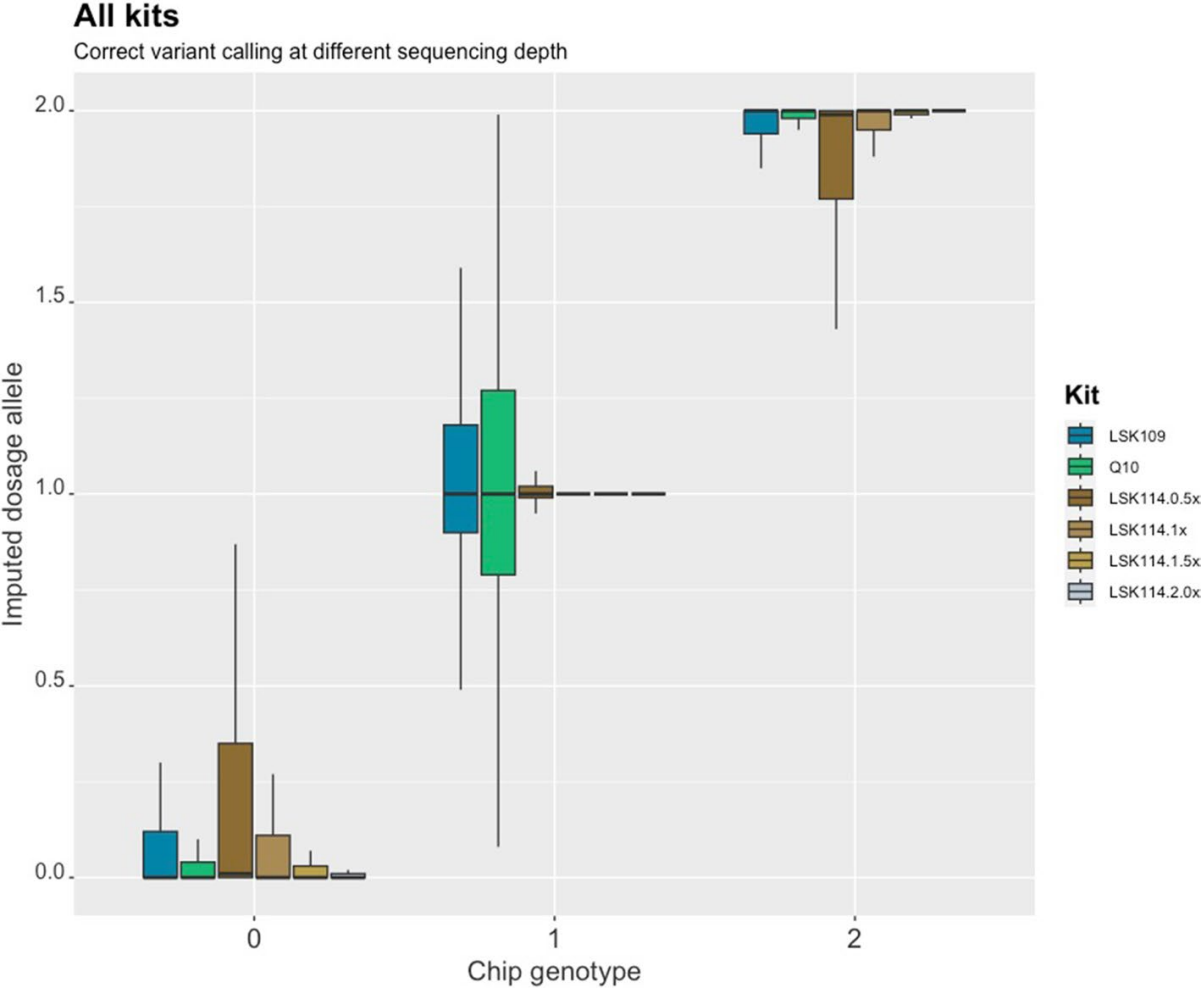
Imputed dosage allele obtained after LPS according to the SNP genotype code. Each kit and sequencing depth (from LSK114) is depicted in different color. Samples from LSK109 (Q20) had average sequencing depth of 0.6 × (0.4 ×)

After imputing called variants to whole genome sequencing, the imputed variants were compared to the genotypes from the SNP array. Figure 3 shows a high degree of concordance between the imputed variant from LPS and the genotype. Lower agreement was observed for heterozygous genotypes when the LSK109 and Q20 kits were used. In these cases, imputation was less accurate. Samples sequenced with the LSK114 kit were accurately imputed, although wider ranges of DA were observed for homozygous SNPs when LPS variants were imputed from sequence depths as low as 0.5 × . In contrats to older chemistry, more accurate DA was imputed from LSK114 even for heterozygote genotypes and at similar sequencing depths ∼ 0.5 × .

**Fig. 4:**
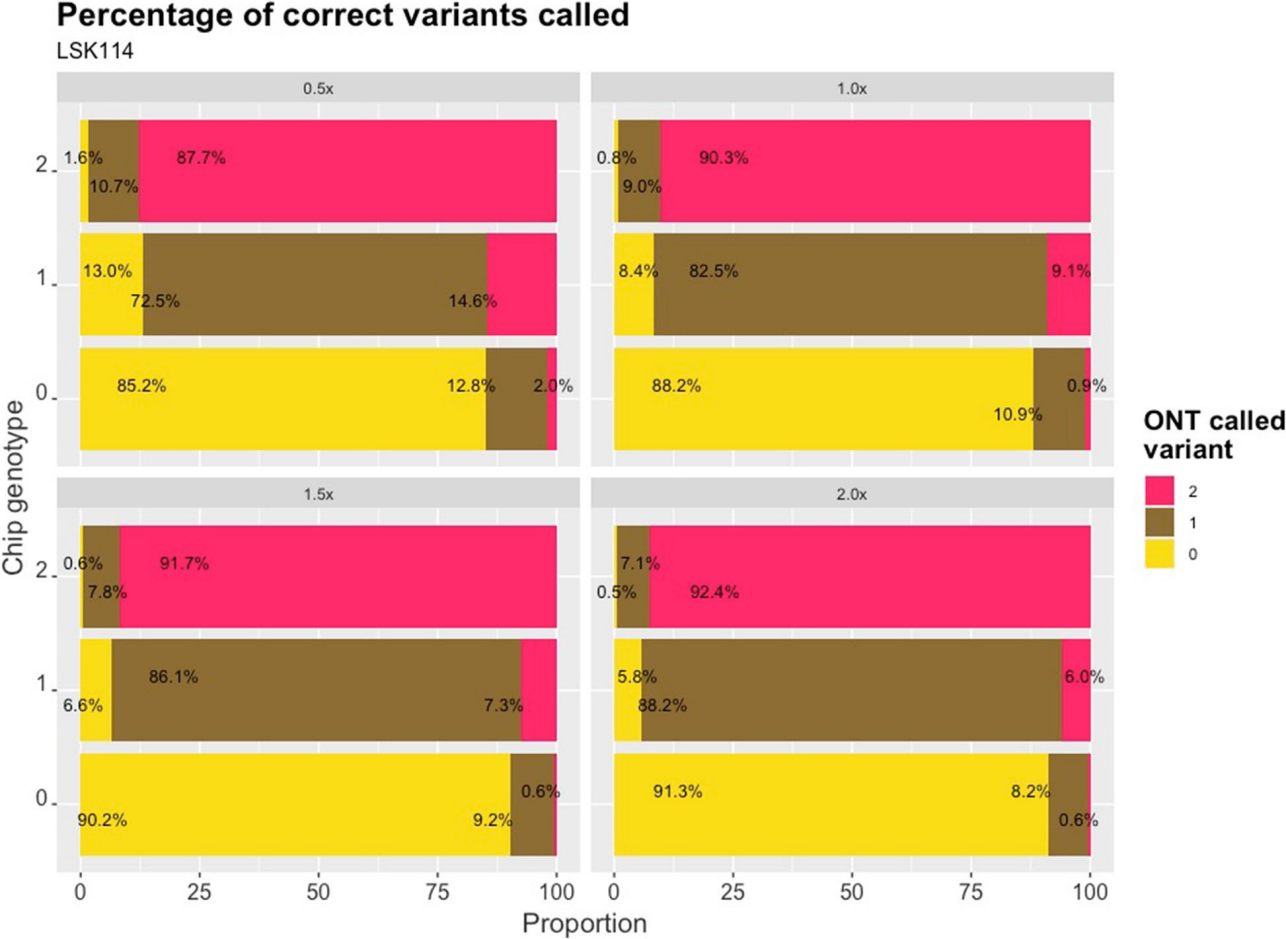
Proportion of called genotypes from LPS for each genotype code from SNP chip (vertical axes). Values are obtained from LSK114 kit at different sequencing depths (0.5 × , 1.0 × , 1.5 × and 2.0 ×)

Commonly, heterozygous genotypes are called for 0.8 ≤ DA ≤ 1.2. The percentage of correct and miscalled genotypes from LSK114 is shown in Fig. 4 at different sequencing depths. A larger amount of correct calls were imputed for homozygous positions ranging from 85.2% at a sequencing depth of 0.5 × to 91.3% at a sequencing depth of 2 × . The mismatches were mainly in only one of the alleles, with ≤ 1% of the sites with both alleles imputed incorrectly. A larger number of errors were observed for heterozygous positions, mainly at a sequencing depth of 0.5 × , with 27.5% of positions being miscalled with one wrong allele. The percentage of mismatches decreased to 11.8% at a sequencing depth of 2 × .

### Closeness between polygenic values estimated from SNP chips and LPS

**Fig. 5:**
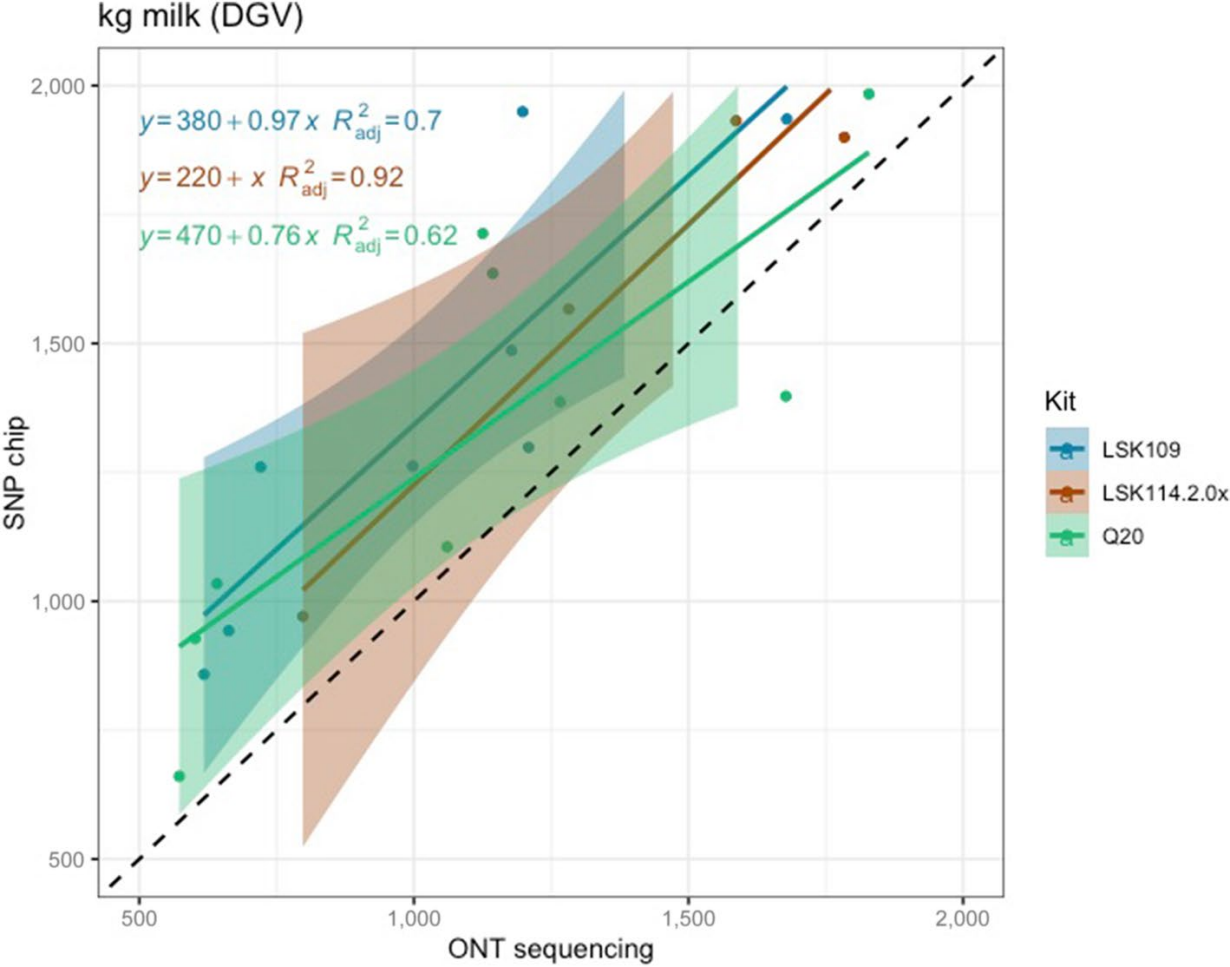
Scatter plot between milk yield DGVs obtained from SNP chips (*y* axis) and genotype-by-LPS (*x* axis) for the different ONT kits evaluated

**Fig. 6:**
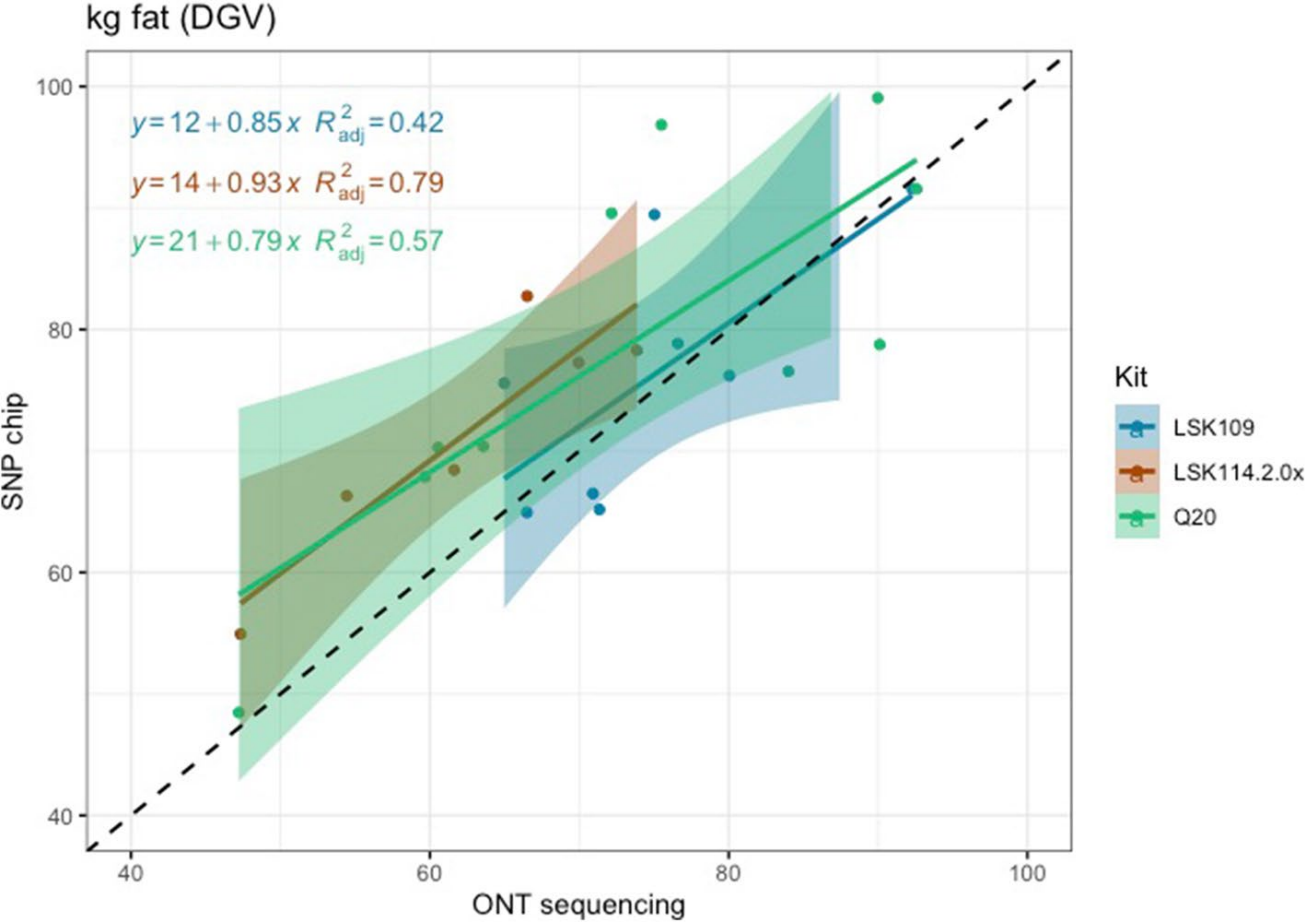
Scatter plot between fat yield DGVs obtained from SNP chips (*y* axis) and genotype-by-LPS (*x* axis) for the different ONT kits evaluated

**Fig. 7:**
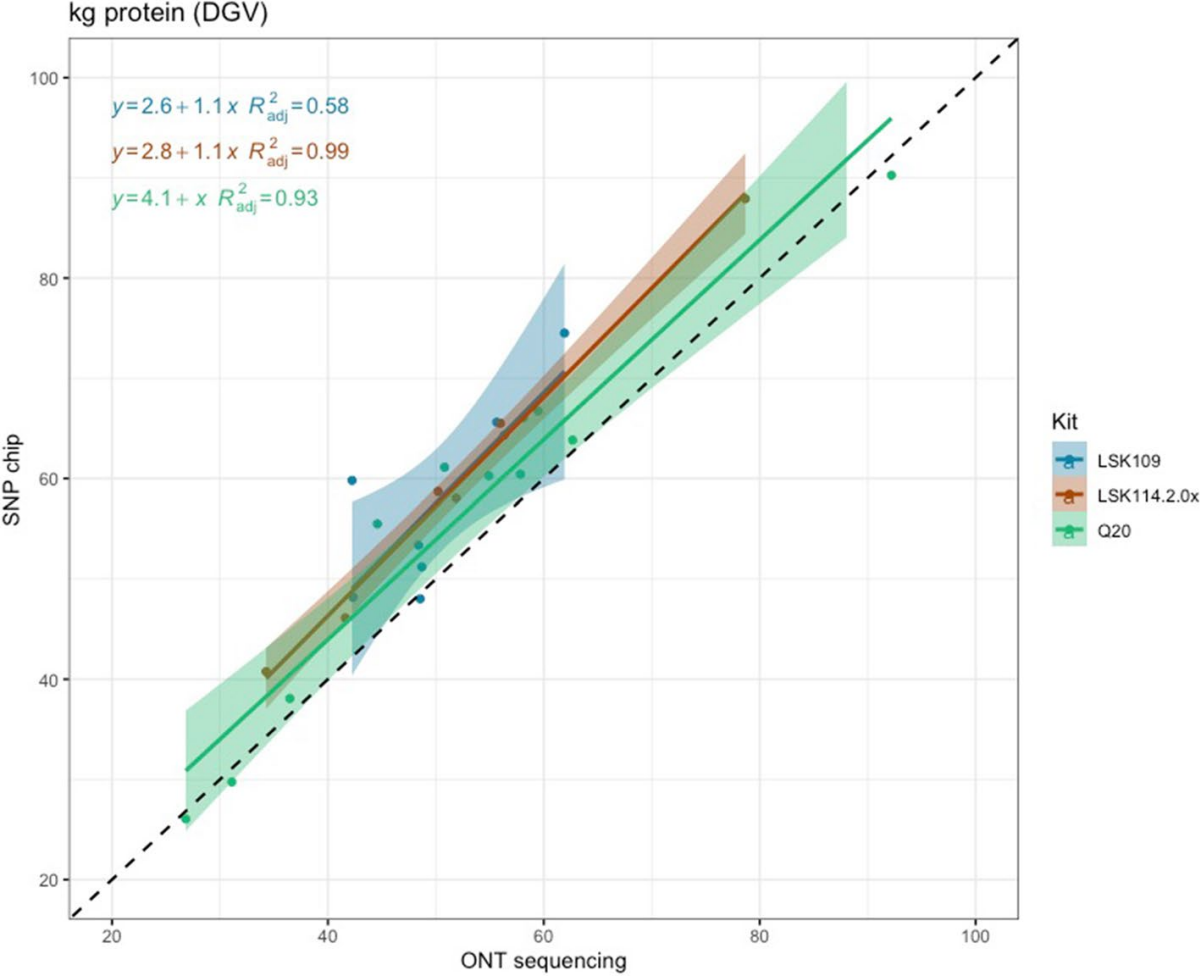
Scatter plot between protein yield DGVs obtained from SNP chips (*y* axis) and genotype-by-LPS (*x* axis) for the different ONT kits evaluated

Pearson correlation between DGV calculated from SNP chips and LPS (all chemistries) was 0.95, 0.84, and 0.95 for MY, FY and PY, respectively. However, the closeness between DGV estimated from LPS was largely influenced by the sequencing kit. LSK114 yielded better R^2^ for all traits (0.92, 0.79 and 0.99 for MY, FY and PY) whereas older chemistry LSK109 showed R^2^ of 0.70, 0.42 and 0.58, respectively (Fig. 5, 6 and 7). The Q20 kit achieved intermediate *R*^2^ values (0.62, 0.57, 0.93 for MY, FY and PY). Regression coefficient was equal to 1 for MY using kit LSK114, and for PY using Q20 kit. Lower agreement between SNP chips and LPS was observed for FY, probably because the dispersion of this trait in the sample set was lower than for the other traits. Table 2 shows the Spearman (rank) correlations between DGVs calculated with SNP chips and LPS. Larger correlations were calculated for LSK114 (0.94, 0.83 and 0.95), suggesting very similar ranking between SNP chips and LPS. Despite of the general strong agreement, the intercept estimates (> 0) showed that ONT sequencing underestimated DGVs for all traits analyzed.

**Table 2:** Spearman correlations between DGVs calculated from SNP chips and nanopore genotype-by-LPS for each sequencing kit and trait evaluated

Kit	Milk yield	Fat yield	Protein yield
LSK109	0.88	0.74	0.62
Q20	0.92	0.85	0.95
LSK114	0.94	0.83	0.95

### Effect of sequencing depth on similarity to SNP chip genotypes

**Fig. 8:**
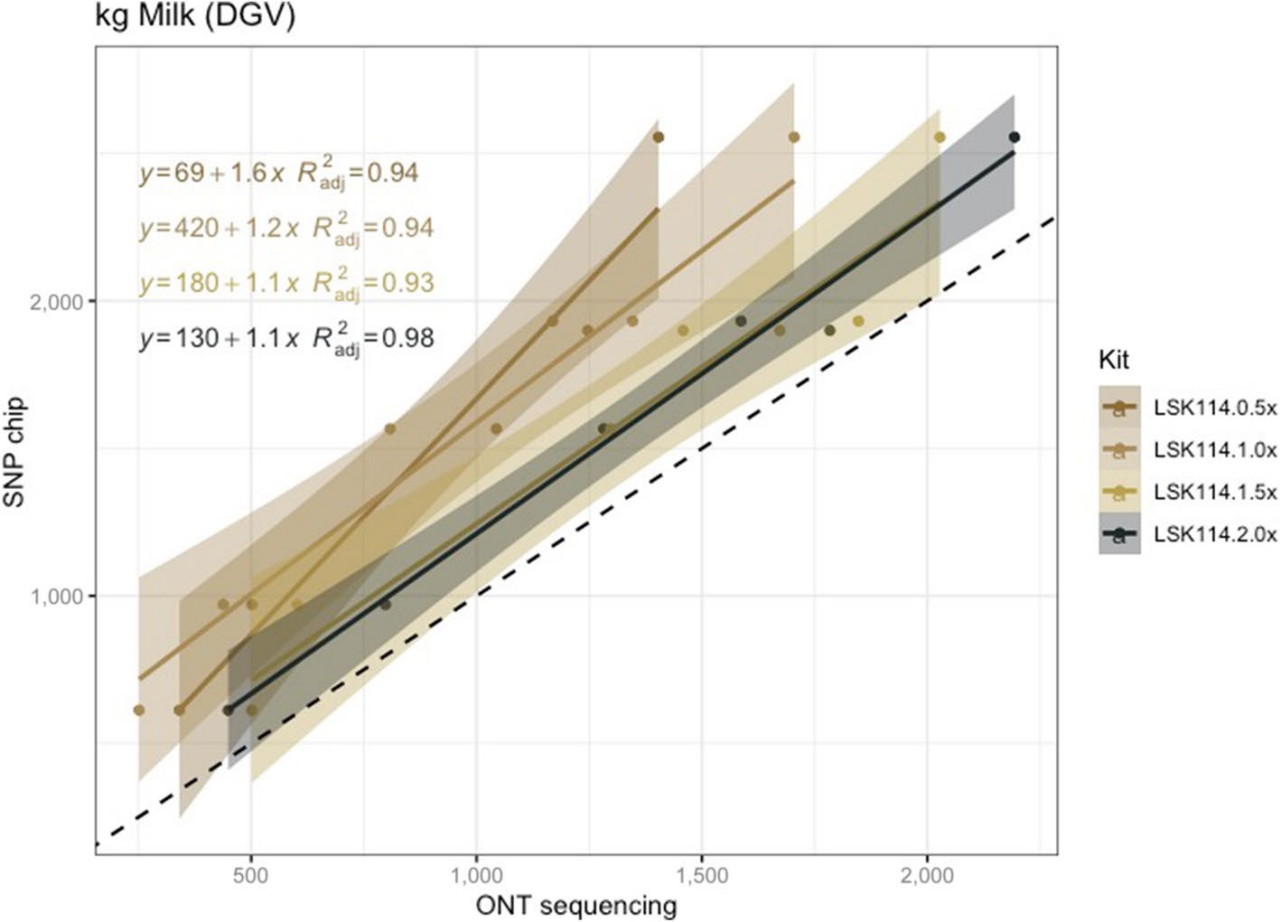
Scatter plot between milk yield DGVs obtained from SNP chips (*y* axis) and genotype-by-LPS (*x* axis) obtained from LSK114 kit at different sequncing depths (0.5 × , 1.0 × , 1.5 × and 2.0 ×)

Since LSK114 was the kit performing best, we hypothesized that this might be due to a larger sequencing depth. Hence, we evaluated whether the higher DGV estimation reliability estimated from LSK114 was due to the higher a sequencing depth in Kit14 or to higher basecalling accuracies. The MY trait was shown here as example, although the same behavior was observed in the other traits (results not shown). The process consisted of randomly selecting a given number of reads for each sample sequenced with the LSK114, to achieve different sequencing depths (i.e., 0.5 × , 1.0 × , 1.5 × and 2.0 ×). Results are depicted in Fig. 8. The *R*^2^ ranged between 0.93–0.94 for sequencing depth < 2 × and 0.98 for sequencing depth of 2 × . Lower sequencing depth resulted in more biased estimates, which may be the reason of the underestimation of the DGVs mentioned above. Larger sequencing depths (2 ×) alleviated this bias in the regression parameter and intercept estimation.

### Detection of modified bases

**Fig. 9:**
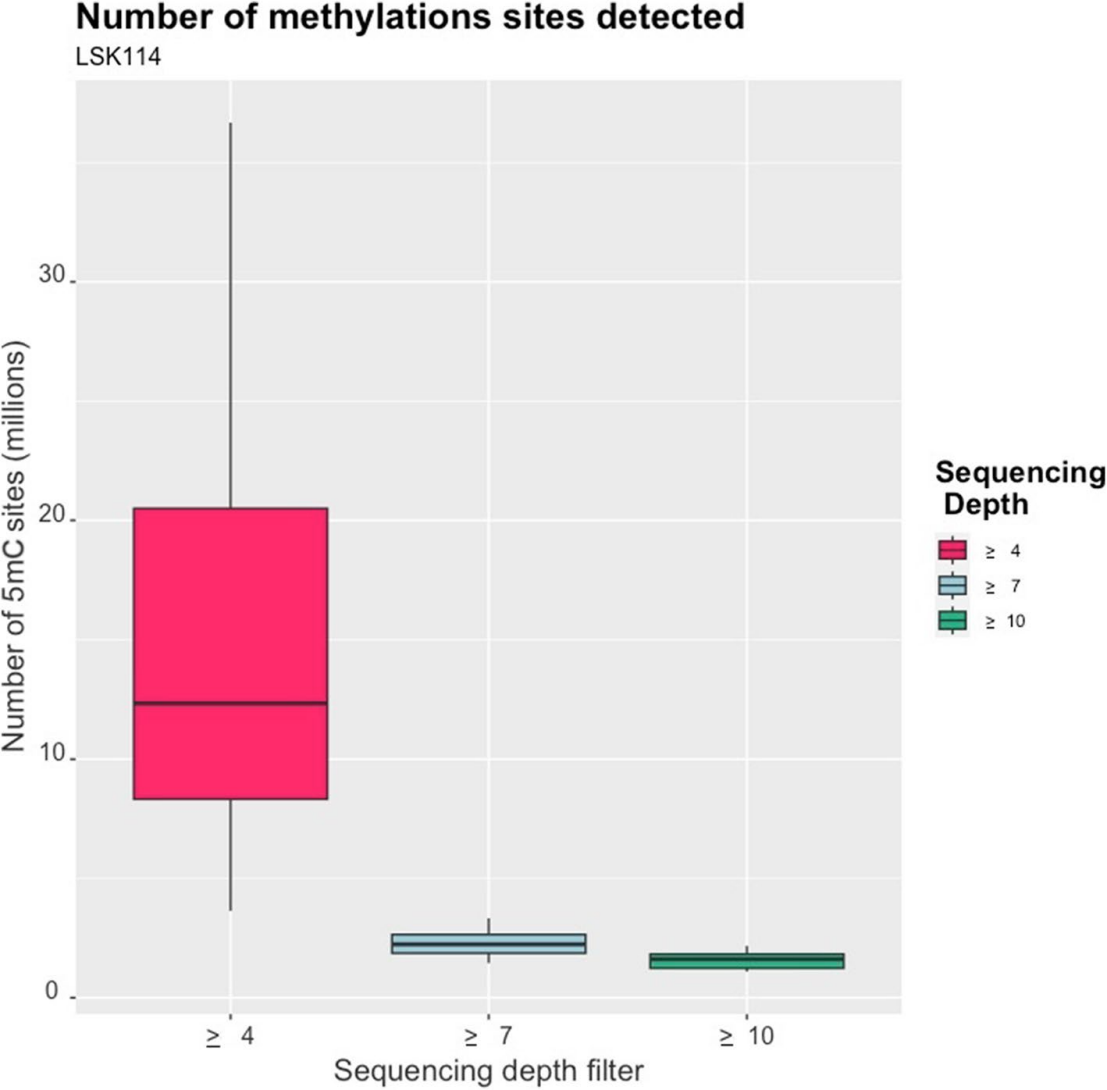
Boxplots for the number of methylated sites obtained from EpiGLowS with LSK114 kit after filtering by sequencing depth ≥ 4 × , ≥ 7 × or ≥ 10 × . Average sequencing depth from EpiGLowS was 2 ×

**Fig. 10:**
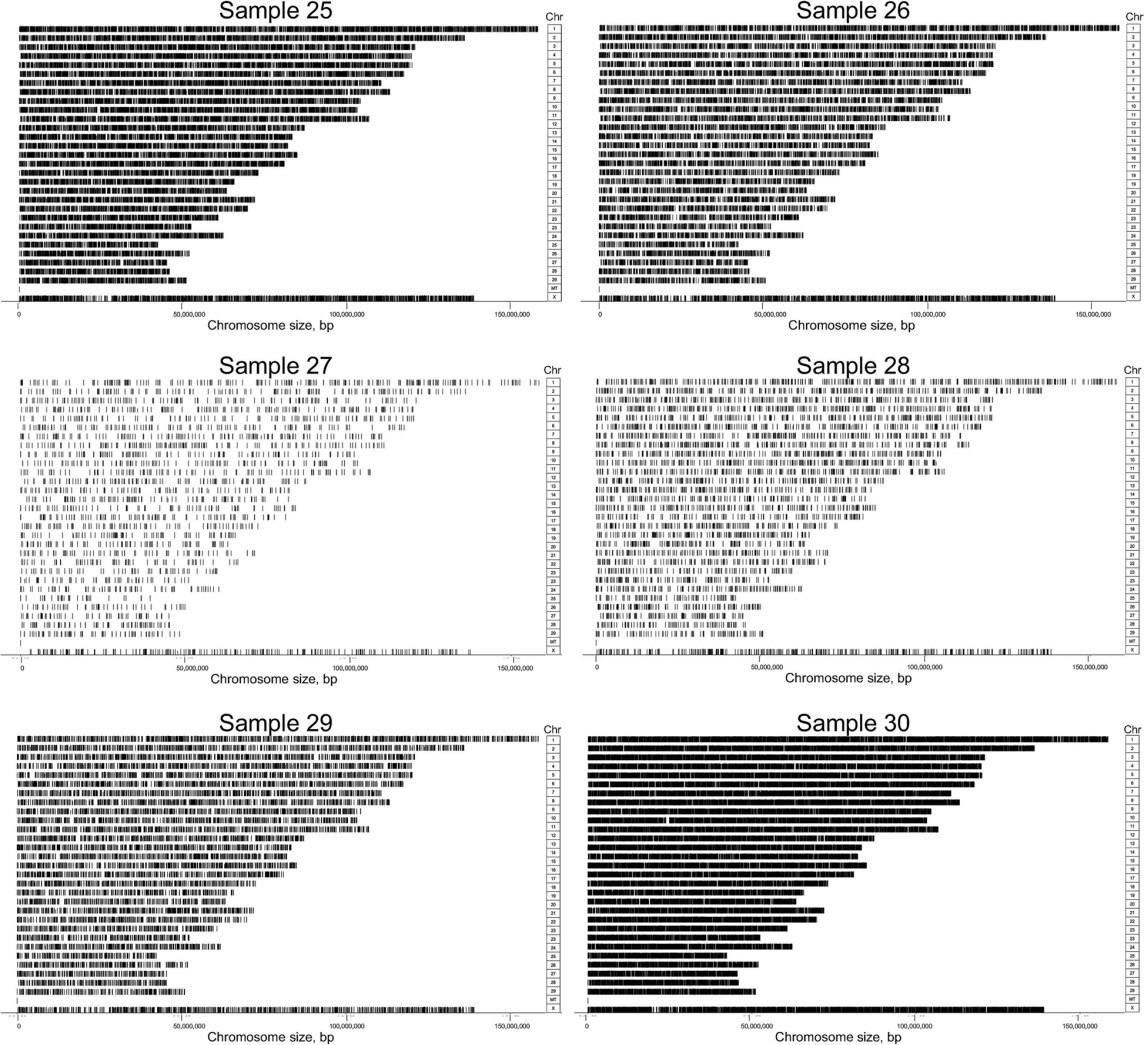
Chromosome-wide methylation sites for each sample sequenced with the LSK114 kit. Positions showed had a coverage ≥ 4 ×

An average of 791 millions 5mC modifications were detected from LSK114 kit using EpiGLowS. However, after filtering for variant coverage ≥ 4 × , the average amount of 5mC detected was 15.7 millions, and decreased to 2.3 and 1.6 millions for variant coverage filters ≥ 7 × and ≥ 10 × , respectively (Fig. 9). In terms of sequencing yield, 5–6 Gb would produce more than 15 million 5mC methylation states at a coverage ≥ 4 × , and at least 1.5 million 5mC sites at coverage ≥ 10 × . We evaluated the differences for coverage filters of 4 × , 7 × and 10 × . A large agreement in the methylation percentage was observed in genomic bins of 500 bp: a correlation of 0.985 was achieved between filters ≥ 4 × and ≥ 10 × , and 0.986 between ≥ 7 × and ≥ 10 × . Figure 10 depicts the genome positions that were methylated for each sample sequenced with the LSK114 kit after filtering for coverage ≥ 4 × . Methylation was detected along the whole genome. Samples with a larger genome coverage and sequencing yield (samples 25, 26 and 30) also showed a larger density of methylated position across the genome at a coverage ≥ 4 × . Those samples with lower genome coverage still showed a genome- wide methylation randomly distributed along the genome, although with a lower density of the methylated sites. Filtering for coverage ≥ 10 × led to much sparser methylation marks, which may impair the number of methylated sites in those samples with lower sequencing depth.

These methylation marks were located mainly in distal intergenic regions, emphasizing the evidence that the genome is pervasively transcribed, and that the majority of its bases are in primary transcripts, including non-protein-coding transcripts [21]. Around 5%–6% of methylated positions were found in promoter regions, and there were little variability in this percentage among samples. Larger variability was found in the percentage of methylated sites found in exons and distal intergenic regions.

**Fig. 11:**
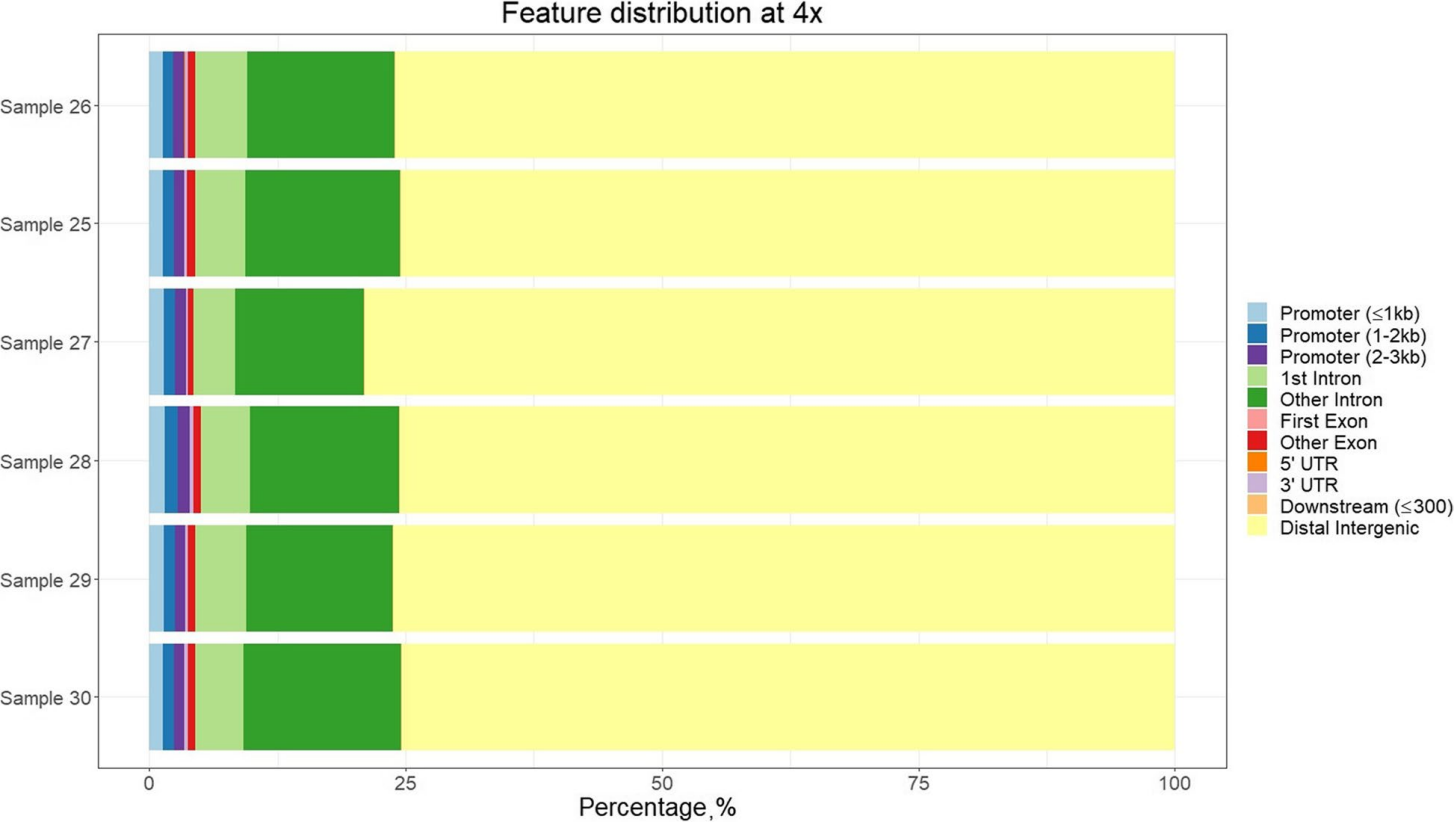
Percentage of methylated genomic regions found for each sample sequenced with the LSK114 kit. Positions showed had a coverage ≥ 4 ×

**Fig. 12:**
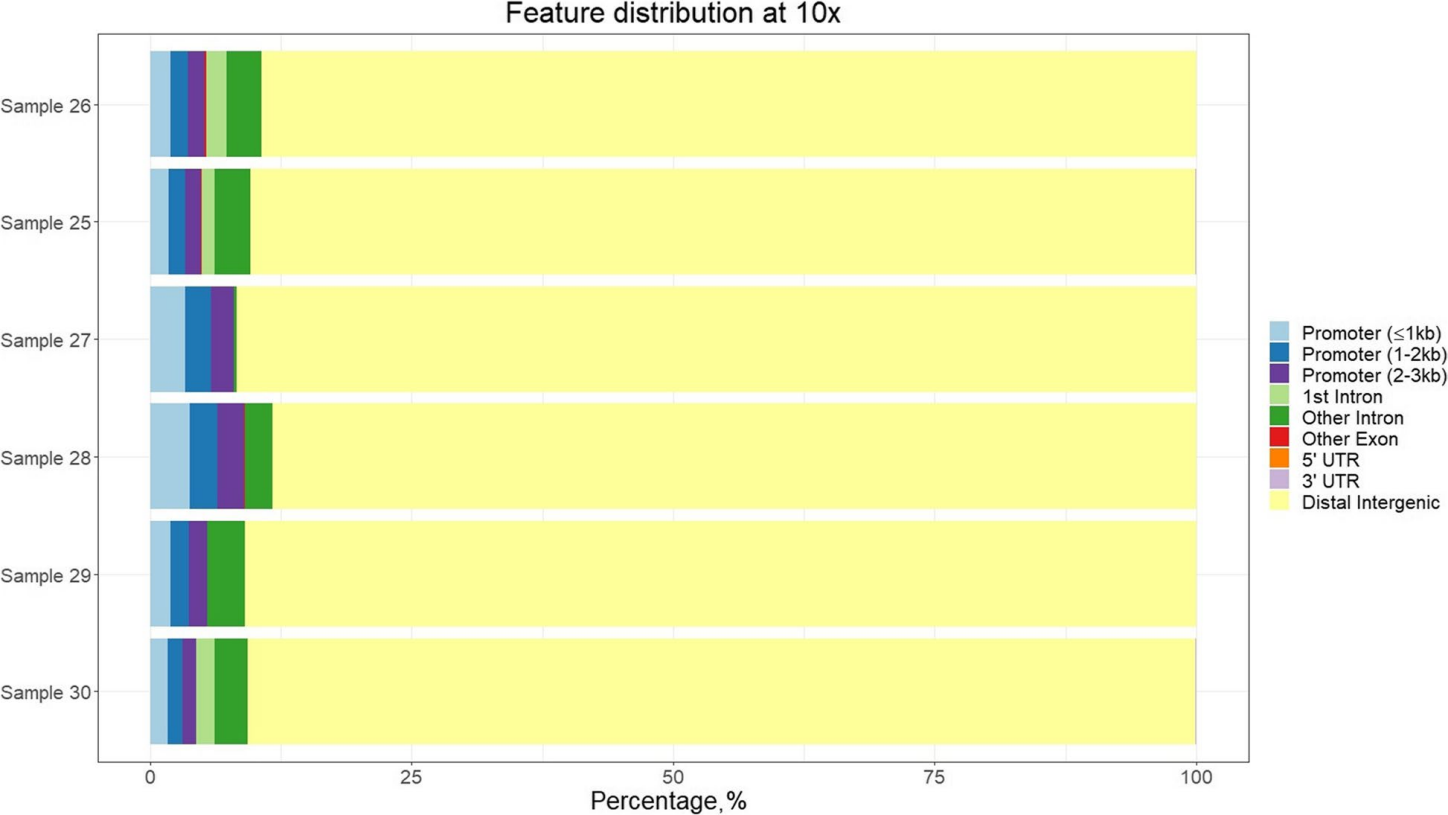
Percentage of methylated genomic regions found for each sample sequenced with the LSK114 kit. Positions showed had a coverage ≥ 10 ×

**Fig. 13:**
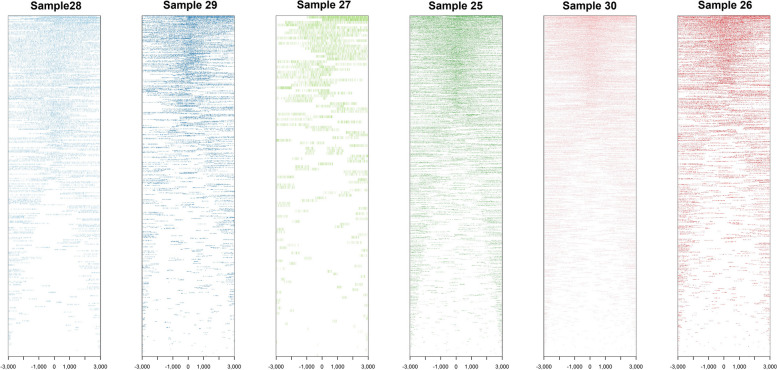
Density of methylated position found for each sample according to their distance to the transcription start site (TSS). Samples were sequenced with LSK114 kit. The plot shows the position left after filtering by sequencing depth ≥ 4 × . Average sequencing depth from EpiGLowS was 2 ×

Filtering for coverage ≥ 10 × led to similar proportions at promoter regions, but a much larger proportion of methylated sites in distal intergenic regions (Fig. 11 and 12). After filtering for coverage ≥ 4 × , the methylation pattern was as expected with a larger density of methylation marks at TSS, and a sudden drop upstream (Fig. 13). It also shows the methylation status near the TSS of known genes. Some genes showed large proportion of methylation marks at or near-by the TSS, which is often maintained upstream during few hundreds bases. Interestingly, other genes showed no methylation at the TSS or nearby, probably because they are constitutive or necessary genes. This deserves further study.

## Discussion

Low pass sequencing has captured interest in later years due to the large amount of information it provides in genetic evaluations and because cost are decreasing fast (e.g., [22, 23]). This is the first study evaluating the similarities between DGVs obtained from traditional SNP chips and different ONT chemistries in an epi-genotype-by-LPS framework, and simultaneously extracting methylation marks, which we called EpiGLowS. It complements previous studies that used ONT sequencing in canola [24] and Australian Droughtmaster [12] with the LSK108 or LSK109 kits. Both studies showed similar basecalling and imputation accuracies as our results. Although those studies used higher sequencing depths and did not detect methylation.

The results from LSK109 and Q20 in our study are comparable to a previous study using ONT sequencing in a genomic selection framework [12]. However, samples in [12] were sequenced with LSK109 and at a much larger depth than in the present study, with an average yield of 22.57 Gb, which is equivalent to > 7 × sequence depth. The DGV accuracies for samples sequenced with LSK114 was similar to those from full coverage in [12]. Based on the results of our study, and in comparison to [12], the new chemistry LSK114 may provide similar results as the old chemistry but with a sequencing depth as low as 2 × . Lamb [12] also showed some prediction biased at very low sequencing depths compared to SNP chip arrays, and this bias was trait-dependent. Although perfect rank agreement with SNP chips was not achieved in our small data set, the closeness obtained is encouraging to pursue new analytical methods with a large data set that may show even larger agreement for genotypes obtained by LPS. Nonetheless, it must be pointed out that the small samples size may negatively impact Spearman correlation, underestimating its true value. Older ONT chemistries posed some bias when used at low sequencing depth. However, the latest LSK114 chemistry provided a high basecalling accuracy that was suitable for breeding value prediction in a genomic selection framework. This limitation may be alleviated by using DA to estimate DGV or polygenic risk scores at a low sequencing depth of 2 × . Very low sequencing depths may still provide high ranking agreement yet with larger bias.

Our study also showed the possibility to simultaneosuly obtain methylation status throughout the genome with a high closeness even at a low sequencing depth, which comes at no extra cost with genotype-by-LPS. This epigenetic information can be used in epigenome-wide association studies to infer association between methylation and phenotypic expression of traits of interest. It can also be included in the mixed models used in quantitative genetics to account for epigenetic variance or to determine the effect of environmental forces on the methylation status [25]. The number of methylated regions and its reliability depends on the coverage filters applied to EpiGLowS. Too stringent filters may underrepresent methylation in promoter regions, since many methylated sites are filtered out. Therefore, a minimum coverage of 4 × may be enough for whole genome methylation, considering that it showed very similar results and high accordance with a coverage filter ≥ 10 × , and kept 10-folds more sites.

EpiGLowS is also appealing in populations where SNP chips are not available or a high density of markers is required (e.g., populations with short linkage disequilibrium range, or studies dealing with rare variants). It may also compensate the cost of obtaining SNP variants and methylation status independently. Additional advantages of ONT sequencing include its portability, and its ability to sequence long DNA fragments to detect structural variants [26]. Despite its affordability, EpiGLowS is still less cost effective than SNP genotyping arrays. Proper multiplexing strategies may contribute to decrease the cost of LPS, while mantaining high accuracies.

It must be pointed out that ONT sequencing has increased the basecalling accuracy through both chemistry improved bioinformatic analyses [27, 28]. Yet, reads with lower basecalling accuracy may introduce error variants in downstream analysis. Thus, new computational tools and more efficient and optimized protocols for sequencing at low depths may be available for more accurate EpiGLowS analysis in the short term. Our study used Beagle 5 for imputation, with a post processing to account for error prone reads. There are other tools that can analyse low coverage sequencing, however they are not specifically designed for error prone long reads [6, 29, 30].

Future strategies might be developed to specifically account for low coverage from long read, and future developments that integrate ONT sequencing in breeding programs may include in-farm LPS for clinical diagnostic or rapid breeding decisions. High performance ONT sequencing platforms, such as Promethion devices, can also complement Illumina platforms to increase the throughput of breeding programs implementing genomic selection. Some computation strategies may be needed to combine short and long reads low pass sequencing within the same population. The results in other populations with a lower number of individuals, and lower genomic prediction accuracies need yet to be tested.

## Conclusions

The latest LSK114 chemistry provided a high basecalling accuracy that was suitable for breeding value prediction in a genomic selection framework with very similar estimated DGVs compared to the tradictional SNP chips. In the future, an increased basecalling accuracy and sequencing yield may lead genotype-by-LPS and EpiGLowS to achieve even higher DGV closeness to SNP genotypes, even at low sequencing depths and at a competitive cost. New research and field application opportunities arise with the proposed genotype-by-LPS in livestock breeding programs and also at evaluating management practices that may impact on the epigenetic status of the animals. Our results showed that EpiGLowS is attractive for research including genomic and epigenomic variants, despite of few limitations such as a lack of full agreement with SNP chip genotypes and low coverage of methylation marks.

## Data Availability

The datasets during and/or analysed during the current study available from the corresponding author on reasonable request.

## Abbreviations

DA: Dosage allele
DGV: Direct genomic value
EpiGLowS: Epi-genotype by low pass sequencing
FY: Fat yield
LPS: Low-pass sequencing
MY: Milk yield
ONT: Oxford Nanopore Technologies
PY: Protein yield
SNP: Single nucleotide polymorphism
TMS: Total mismatch score
TSS: Transcription start site
